# Buildability and Mechanical Properties of 3D Printed Concrete

**DOI:** 10.3390/ma13214919

**Published:** 2020-11-02

**Authors:** Changbin Joh, Jungwoo Lee, The Quang Bui, Jihun Park, In-Hwan Yang

**Affiliations:** 1 Department of Infrastructure Safety Research, Korea Institute of Civil Engineering and Building Technology, Goyang, Gyeonggi 10223, Korea; cjoh@kict.re.kr (C.J.); duckhawk@kict.re.kr (J.L.); 2 Department of Civil Engineering, Kunsan National University, Kunsan, Jeonbuk 54150, Korea; tqbui93@gmail.com (T.Q.B.); jhpark3@kunsan.ac.kr (J.P.)

**Keywords:** 3D concrete printing, buildability, interlayer interval time, lateral support, mechanical properties

## Abstract

Recently, 3D concrete printing has progressed rapidly in the construction industry. However, this technique still contains several factors that influence the buildability and mechanical properties of the printed concrete. Therefore, this study investigated the effects of the nozzle speed, the interlayer interval time, the rotations per minute (RPMs) of the screw in the 3D printing device, and the presence of lateral supports on the buildability of 3D concrete printing. In addition, this paper presents the results of the mechanical properties, including the compressive, splitting tensile, and flexural tensile strengths of 3D printed concrete. The buildability of 3D printed structures was improved with an extended interlayer interval time of up to 300 s. The printing processes were interrupted because of tearing of concrete filaments, which was related to excessive RPMs of the mixing screw. The test results also showed that a lateral support with a wide contact surface could improve the resistance to buckling failure for 3D printed structures. The test results of the mechanical properties of the 3D printed concrete specimens indicated that the compressive, splitting tensile, and flexural tensile strengths significantly depended on the bonding behavior at the interlayers of the printed specimens. In addition, although metal laths were expected to improve the tensile strength of the printed specimens, they adversely affected the tensile performance due to weak bonding between the reinforcements and concrete filaments.

## 1. Introduction

The 3D printing technique has become promising for prefabricated structures because of its outstanding flexibility in both architectural and structural designs [1]. Without the facilitation of formwork, structures with various shapes can be built quickly by using this technique [2,3,4]; thus, 3D printing is being rapidly applied in the building industry [5,6,7,8]. In addition, the 3D printing technique also reduces the cost and amount of materials for construction compared to those for traditional on-site construction methods [9,10,11,12].

Although the 3D printing method is considered a promising technique, some factors still influence the printing performance. For example, the printing performance is strongly dependent on the fresh properties of concrete, such as its rheology and green strength [13,14,15], and the printing parameters, such as the nozzle speed, nozzle height, interlayer interval time, and extrusion rate [16,17,18,19]. These factors also influence the mechanical properties of 3D printed concrete in the hardened state. Therefore, it is important to investigate the buildability for the printing of concrete structures and the mechanical properties of hardened concrete.

Panda et al. [18] investigated the effect of different nozzle speeds on the properties of fresh concrete used for 3D printing. They pointed out that the optimum printing speed would ensure a constant bead width of the layers throughout the printing process, and the bonding strengths of the printed specimens slightly reduced with increasing nozzle speed. Kruger et al. [20] developed a design model for 3D concrete printing and predicted the printing speed to prevent the failure of the structure under the given conditions.

The effect of the interlayer interval time (delay time) during 3D concrete printing also has a significant influence on the buildability as well as the interlayer bonding strength of filaments. According to Le et al. [21] and Sanjayan et al. [22], the interlayer bonding strength decreased with increasing interlayer interval time. In addition, they recommended that it was necessary to optimize the interlayer interval time to develop the green strength of fresh filaments to prevent significant deformation or collapse and ensure an acceptable bonding strength between printed layers.

Moreover, the printed structure may collapse suddenly by buckling during printing; the buckling behavior depends on the geometry and support conditions of the structure. Thus, lateral support or reinforcement is needed [23,24]. For example, steel wire mesh reinforcement improved the flexural strength of printed specimens [25], although there were obvious limitations in the printing process and the design of the nozzle for the reinforcement.

In addition, the anisotropic strength of 3D printed concrete is a concern because the printing direction significantly affects the material properties of 3D printed concrete in the hardened state [15,16,21,26,27,28]. Nerella et al. [16] investigated the compressive and flexural tensile strengths of 3D printed specimens in three different directions. The layer directions slightly affected the compressive strength but significantly influenced the flexural tensile strength of the printed specimens. However, Wolfs et al. [27] reported that the effect of the layer directions on the mechanical properties of 3D printed concrete was insignificant based on the measurements of the compressive, splitting tensile, and flexural strengths in three different directions for the printed specimens.

Based on previous studies, the aim of this study was to investigate the buildability of 3D printed concrete during printing and the mechanical properties of the 3D printed concrete in a hardened state. The effects of the nozzle speed, interlayer interval time, and presence of lateral support on the buildability of the 3D concrete printing were investigated. To investigate the mechanical properties of 3D printed concrete, compressive, splitting tensile, and flexural tensile tests in three different loading directions were carried out. Finally, the mechanical properties of the monolithic specimens and printed specimens were compared.

## 2. Experimental Program

### 2.1. Printing Method

**Figure 1:**
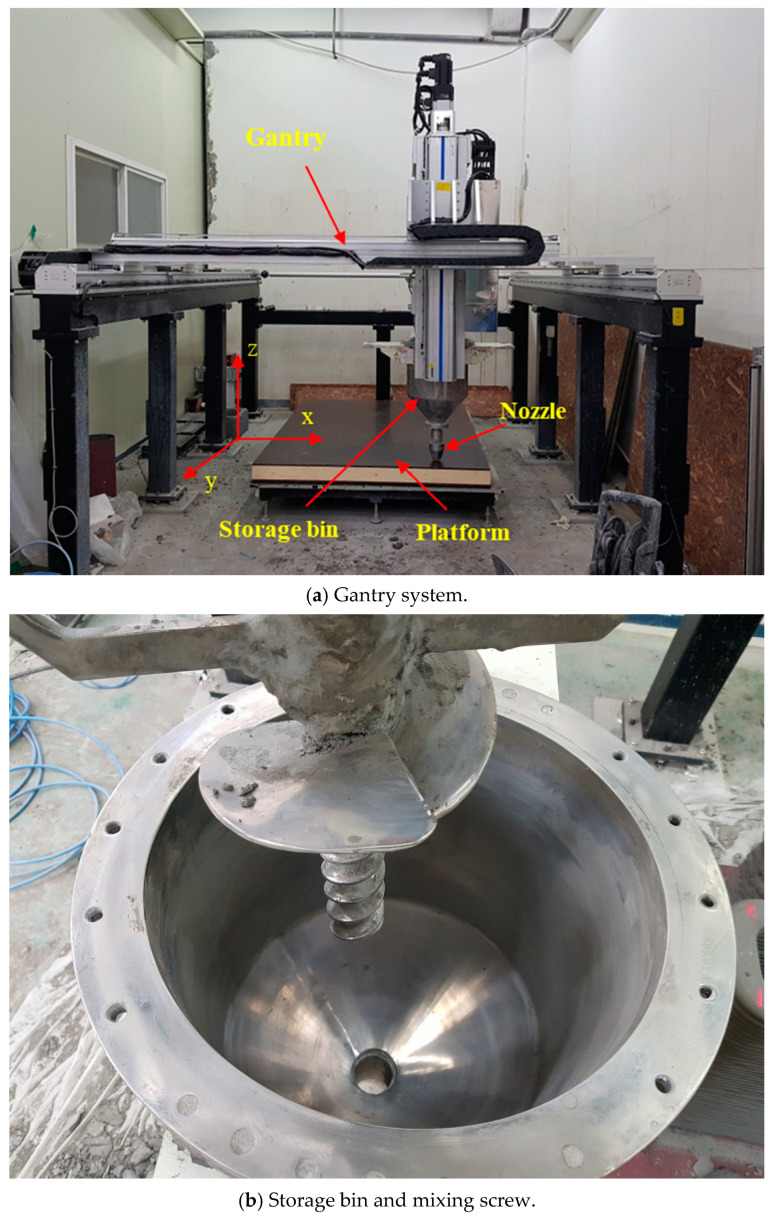
3D printing equipment.

Currently, several printing techniques have been applied in building construction, such as contour crafting [29], Apis-Cor [30], concrete printing [31], and D-shape [32], which are based on additive manufacturing techniques. The concrete printing technique, which has outstanding control of internal and external geometries, was applied in this study. This system consisted of four major parts: a computer controller, a gantry system, a nozzle part, and a platform, as shown in Figure 1a. The nozzle part was carried on by the gantry system on two parallel rails, so it could move in both the x and y directions, as shown in Figure 1a. Additionally, the nozzle elevation could be adjusted in the z direction during the printing process. A nozzle with a diameter of 40 mm was attached to a storage bin with a volume capacity of 0.05 m^3^, as shown in Figure 1b. A vertical screw rod rotating during the printing process was equipped inside the storage bin to maintain the extrusion and workability of the mortar filament.

### 2.2. Printed Structures and Printing Process

To investigate the buildability and mechanical properties of 3D printed concrete, ten concrete structures were printed, as listed in Table 1. All structures were printed into rectangular wall shapes from the top view. The dimensions of ten structures are also shown in Table 1. The ten 3D printed concrete structures were categorized into three groups.

Three structures, namely, S1, S2, and S3 in Group 1, were printed to investigate the effect of the interlayer interval time and rotations per minute (RPMs) of the mixing screw on the buildability of the structures. Accordingly, the three structures in Group 1 had the same shapes and dimensions but different interlayer interval times or RPMs of the mixing screw.

**Figure 2:**
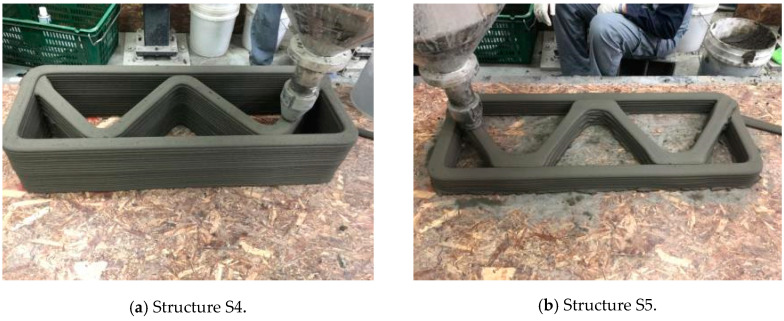
Printing of structures S4 and S5.

Three structures, namely, S4, S5, and S6 in Group 2, were printed to investigate the effect of lateral supports inside walls on the buildability of the structures. Accordingly, wall members of the three structures were supported laterally by members with zigzag shapes inside walls. Structures S4 and S5 had the same rectangular wall dimensions, but the details of the connected parts between the wall and lateral supports were different from each other. For structure S4, the wall and lateral supports were connected to the wall by point contact, as shown in Figure 2a, and for structure S5, the wall and lateral supports were connected in parallel with a width of 100 mm, as shown in Figure 2b. Details of the dimensions of structures S4 and S5 can be found in Table 1. For structure S6, the wall and lateral supports were also connected in parallel with a width of 100 mm.

**Figure 3:**
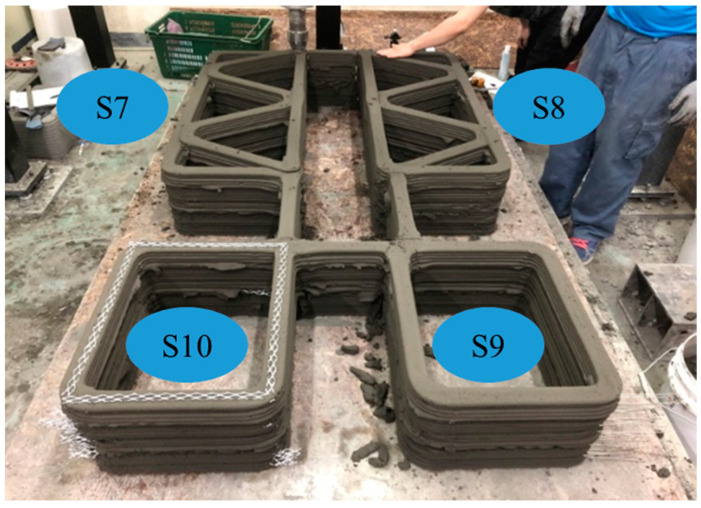
Simultaneous printing of structures S7, S8, S9, and S10.

**Figure 4:**
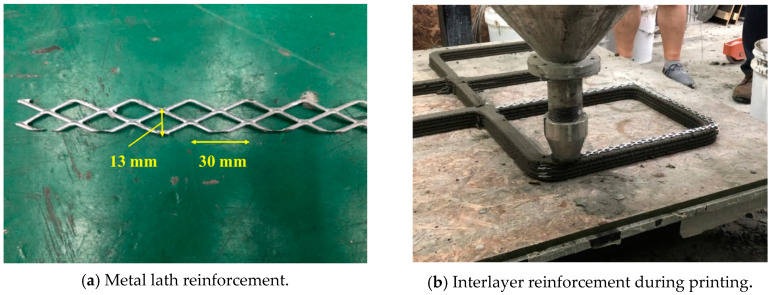
Interlayer reinforcement from metal laths (Structure S10).

Four structures, namely, S7 through S10 in Group 3, were printed to investigate the effect of the interlayer interval time on the buildability of the structures and mechanical properties in the hardened state of the 3D printed concrete. Among these four structures, structures S7 and S8 were duplicates, and structures S9 and S10 were also duplicates except for the interlayer reinforcement. The four structures were printed with the same printing process, as shown in Figure 3. Accordingly, the interlayer interval time of the four structures in Group 3 was 300 s, which was much greater than those of the other structures in Groups 1 and 2. In addition, to study the effect of the reinforcement between the layers on the strength of the 3D printed concrete, metal laths were used as the interlayer reinforcement. The metal lath reinforcement used in this study was made of aluminum. The nominal yielding strength of the metal laths was 270 MPa, and the laths had the shape of an extended parallelogram, as shown in Figure 4a. The lengths of the short and long diagonal distances were 13 and 30 mm, respectively, and the thickness was 2 mm. A metal lath was attached to the interfaces between the layers in structure S10, as shown in Figure 4b. No special surface treatment method was used to increase the bonding strength between the printed concrete and metal laths. The metal laths were laid manually over the printed layer; then, a new layer was repeatedly printed over the metal laths without any artificial interlayer treatment. Therefore, the metal laths were bonded between the printed concrete by the self-weight of the metal laths and printed concrete. After the buildability of the four structures in Group 3 was investigated, the walls of the hardened structures were cut into cubic and prismatic specimens for compressive, splitting tensile, and flexural tensile tests.

### 2.3. Mixture

The mixture used for the 3D printing process is given in Table 2. Ordinary Portland cement (OPC, Asia cement, Seoul, Korea) with a density of 3.14 g/cm^3^ was used for the mixture. Additionally, two types of cementitious materials, namely, silica fume (SF, Asia cement, Seoul, Korea) and class C fly ash (FA, Asia cement, Seoul, Korea), were added to the mixture. To improve the extrusion and bonding properties, SF with a SiO_2_ content of 91.3% was used as a supplementary cementitious material. Class C FA used in the mixture had a density of 2.26 g/cm^3^ and a loss on ignition of 2.8%. Sand with a size range from 0.16–0.2 mm was selected to ensure good extrusion during the printing process. To increase the concrete filament extrusion under the low water-binder ratio of 0.29, a high-performance water-reducing agent (HWRA, Dongnam, Seongnam, Korea) was added to the mixture. Finally, the mixture included a viscosity agent to prevent segregation of the materials and enhance the viscosity of the mixture.

### 2.4. Material Properties in the Fresh State

The repeatability of the process is a critical requirement for 3D printing technology. The width, height, and material characteristics of the printed structures should remain almost the same for each printing process. To achieve repeatable printing in this study, the nozzle height and speed, concrete pumping rate, and RPMs of the mixing screw were controlled by an accurate printing device. Moreover, the test structures were printed on a stable platform. A nozzle height of 10 mm from the existing printed layer was maintained during the printing process. In addition, to control the quality of the mixture, the properties of the materials in the fresh state were measured.

The rheological properties of printing concrete primarily affect extrusion. Therefore, the fluidity and extrusion rate of concrete were measured before printing the structures. The fluidity of the concrete filament was estimated by a flow table test according to ASTM C 230 [33]. The extrusion rate was evaluated by measuring the weight of extruded concrete in one minute. The test results showed that the diameter of the mortar on the flow table was 148 mm and the extrusion rate was 0.133 kg/s. In addition, the rheological yield stress of the concrete was measured by using a rheometer (Brookfield DV-III). The yield stress was approximately 413 Pa, and the plastic viscosity was 19.6 Pa-s.

The measurement of early-age mechanical strength is one of the possible ways to find the stiffness of printing materials. Green strength measurement by a uniaxial unconfined compression test on cylindrical samples has been found to be useful in concrete printing applications. Voigt et al. [34] found that the green strength had a close relationship with the microstructure of the concrete. The test results of Panda et al. [35] showed that green strength developed linearly with time for the initial 30–40 min and then exponentially increased.

For green strength measurement, the specimen preparation and testing process should be designed to minimize unintentional breakdown of the thixotropic behavior of the concrete [35]. Moreover, the vertical and lateral deformation of the sample should be measured using a special device. However, in this study, the green strength at early ages was not measured because there were some difficulties in securing a testing machine with a small loading rate and in capturing dynamic measurements of the changing cross-section of concrete samples.

## 3. Buildability in the Fresh State

The critical criterion for fabricating concrete structures via 3D printing is the vertical deposition of filament layers. The buildability is a significant parameter to evaluate the printable performance of concrete filaments. The buildability refers to the resistance of the deposited fresh concrete to deformation during construction and the ability of the concrete to retain its extruded shape [13]. The 3D printability of concrete, especially the buildability, depends on material parameters (the rheology, green strength, and stiffness of the fresh concrete) and printing parameters (the interlayer interval time incorporated by the nozzle speed, nozzle height, RPMs of the screw, and structural stability parameter) [35,36]. This study focused on the effect of the printing parameters and structural stability parameter on the buildability of 3D printed concrete. The printing parameters included the interlayer interval time and RPMs of the screw, and the structural stability parameter included the presence of lateral supports.

### 3.1. Interlayer Interval Time

**Figure 5:**
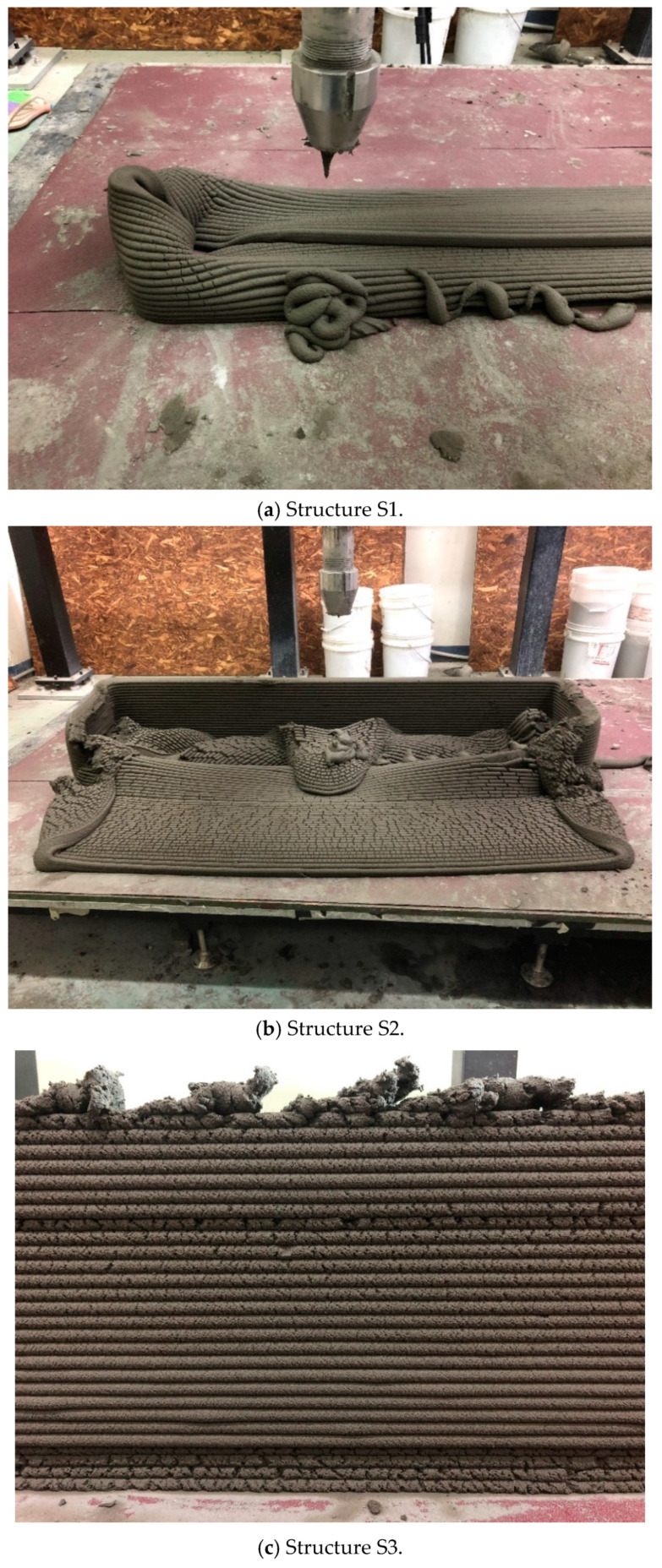

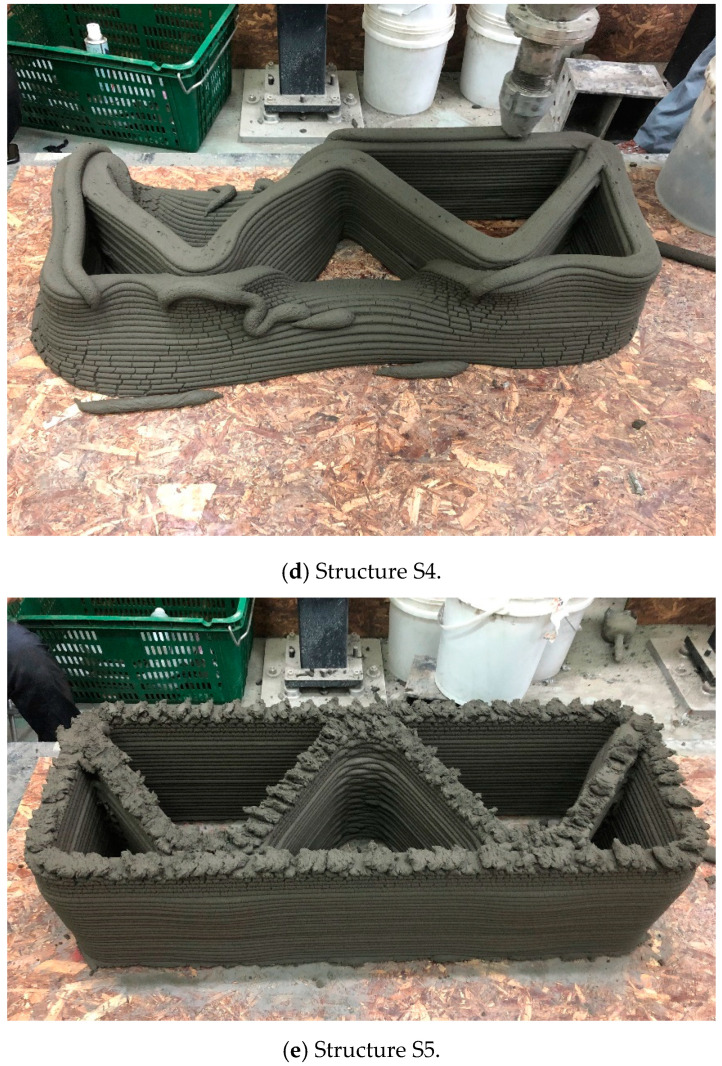

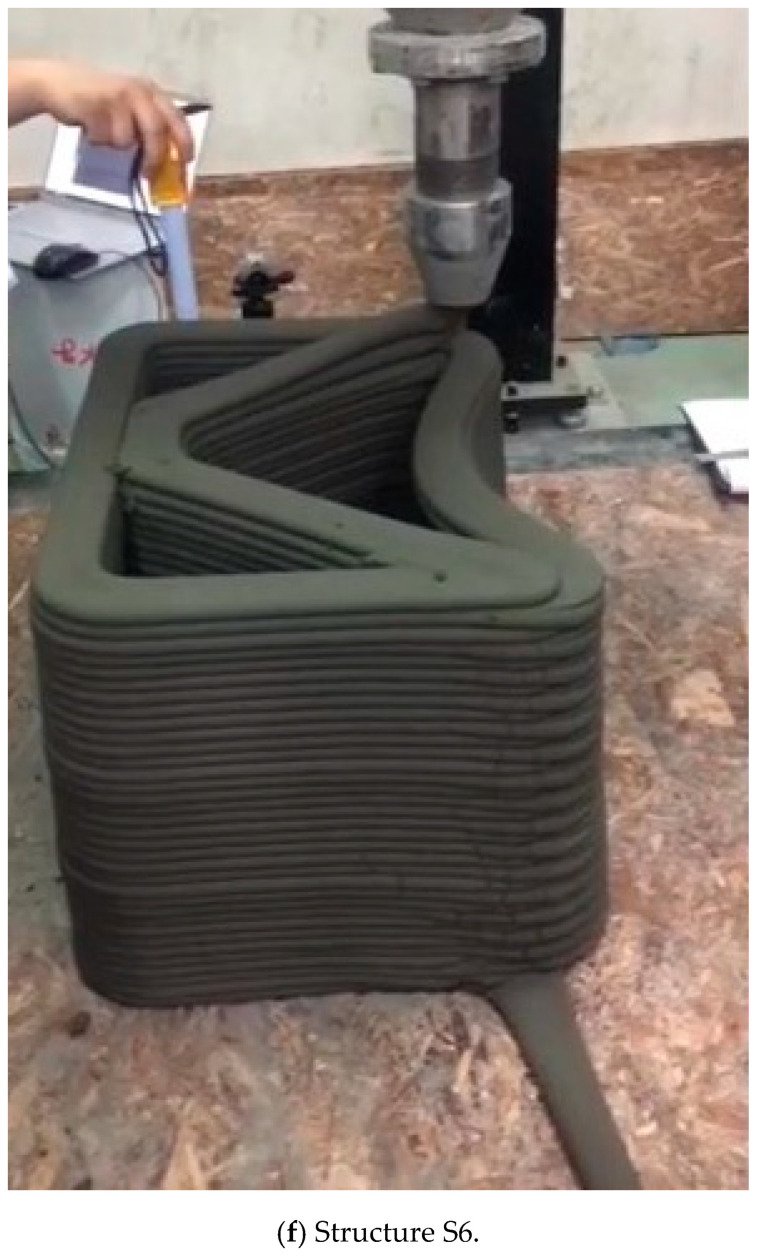
Ultimate state of structures S1 through S6.

The buildability of 3D printed concrete structures was investigated based on the layer deposition process. Among the six S1 through S6 structures, S1, S2, S4, and S6 collapsed suddenly, as shown in Figure 5a,b,d,f, respectively. The collapse occurred at the wall members with the maximum length between the supports where the lateral displacement was prevented. Moreover, structures S3 and S5 showed tearing of the concrete filaments at the uppermost layer, as shown in Figure 5c,e, respectively. The printing of the structures was stopped when tearing of the concrete filaments was observed.

To investigate the influence of the interlayer interval time on the buildability of the 3D printed concrete, three different nozzle speeds of 50, 80, and 100 s and different structural shapes were applied to print the concrete structures, as shown in Table 1.

First, structures S1, S2, and S3 were printed with the same dimensions of 1500 mm × 300 mm along the centerline of the wall but with different nozzle speeds. Due to the different nozzle speeds, structure S1 was printed with an interlayer interval time of 36 s, while structures S2 and S3 were printed with an interlayer interval time of 45 s. The maximum number of printed layers in structures S1, S2, and S3 were 19, 49, and 29, respectively. The maximum number of printed layers upon the collapse of structure S1 was much lower than those of structures S2 and S3. Therefore, the test results showed that the buildability of the 3D concrete printed structures increased as the interlayer interval time increased. According to previous studies [18,21,27,37,38,39], an increased interlayer interval time could provide sufficient time for the early strength development of the low layers in the 3D printed concrete structures and thus improve the buildability of the structures. On the other hand, extending the interlayer interval time could reduce the bonding strength between the layers.

Structures S2 and S3 were printed with the same interlayer interval time but with different RPM values of the screw in the storage bin during printing of the structures. The printing of structure S3 was stopped when tearing of the filaments in the structure in the top layer was observed, as shown in Figure 5c. When tearing of the filaments in structure S3 was observed, the maximum number of printed layers was 29, which is lower than that of structure S2. This was because the RPM value of the screw during printing of structure S3 was higher than that of structure S2, as shown in Table 1. The high speed of the screw in the storage bin might have caused excessive friction heat between the screw and concrete filaments and thus decreased the fluidity of the concrete. Finally, excessive friction heat affected the extrusion of the concrete, and thus, the printing process was stopped when concrete filaments were torn. Therefore, the test results in this study indicated that the RPM value of the screw could affect the buildability when the printing device in a storage bin containing a mixing screw is used for 3D concrete printing.

The buildability of structures S5 and S7, which had similar geometries, was also compared to investigate the buildability of 3D printed concrete at different interlayer interval times. Structure S7 with an interlayer interval time of 300 s was printed up to 52 layers, whereas structure S5 with an interval time of 57 s was printed up to 28 layers. The comparison also showed that an increased interlayer interval time favorably affected the buildability of the printed concrete.

The different interlayer interval times in structures S6 and S9 were compared because the two structures had the same long wall length of 500 mm between lateral supports. Structure S6 was printed with an interval time of 30 s, while the interlayer interval time of structure S9 was extended to ten times that of structure S6. Structure S6 was stacked up to 33 layers and then collapsed by buckling along the long wall. Moreover, structure S9 was printed up to 52 layers, and then printing of the structure was stopped because it was to be used for investigating the mechanical properties of the concrete in the hardened state.

Therefore, a comparison of printed structures with interlayer interval times ranging from 36 to 300 s showed that an increased interlayer interval time favorably affected the buildability of the concrete. The interlayer interval time of the printing process influenced the green strength of the 3D printed concrete. If the interlayer interval time was too short, the green strength of the 3D printed concrete was not high enough to support the upper layers, which might lead to early collapse of the structure, and vice versa.

### 3.2. Lateral Supports

The 3D printed concrete structures in Group 1 in this study failed by buckling, so it was important for the 3D printed concrete structures to resist buckling. The lateral supports printed inside the structure could enhance the resistance to buckling failure along the long wall of the structures. Two types of zigzag lateral supports were printed inside structures S4 and S5, as presented in Figure 2.

Structure S4 contained lateral supports that were printed inside the structure and connected to the long wall by a contacting point. Structure S5 contained lateral supports inside the structure, the arrangement of which was similar to that in structure S4, but they were connected to the long wall by a contact with a width of 100 mm.

The test results showed that structure S4 was printed up to 22 layers and then collapsed by buckling failure along the long wall. Moreover, 28 layers were stacked in structure S5, and then the printing process was interrupted because of the tearing of the concrete in the top layer, as shown in Figure 5e. Both structures S4 and S5 were printed with the same nozzle speed and RPM value of the mixing screw. This implied that the types of connections of the lateral supports to the wall affected the buckling resistance and thus the buildability of the printed structures. By incorporating the lateral supports inside the structure, the long wall of the printed structure could be divided into several spans along the wall. Depending on the types of connections of the lateral supports to the wall, the long wall of structure S4 was divided into two spans with lengths of approximately 500 mm, whereas the long wall of structure S5 was divided into two spans with lengths of approximately 400 mm. This difference in the span length might result in a higher resistance to buckling for structure S5 than that for structure S4.

Similarly, structure S6 was printed with lateral supports that were connected to the long wall by contact points with a width of 100 mm. Structure S6 was stacked up to 33 layers prior to collapse, although it was printed with a shorter interlayer interval time of 30 s than that of 55 s for structure S4. The number of printed layers in structure S6 was higher than that in structure S4.

Structure S6 failed due to buckling because this structure was printed with a short interlayer interval time.

Therefore, the zigzag-shaped lateral supports printed inside the structure, especially with long connecting widths, could increase the resistance to buckling failure in 3D printed concrete structures. The test results in this study are in good agreement with those of a previous study [36], where 3D printing reinforced by zigzag internal layers inside the structure improved not only the buildability of the printed structure but also the resistance to buckling of the printed hardened structure.

A numerical model was developed to predict different failure modes based on the material properties of the concrete in the fresh state and on the failure criterion by Wolfs et al. [40]. According to their study, the failure of 3D printed concrete wall structures was categorized into elastic buckling and plastic collapse. The parametric model used to predict the structural failure mode included the main printing process parameters, which were the curing characteristics of the printing material, the printing speed, the geometrical characteristics of the printed structure, its dead weight, its heterogeneous strength and stiffness characteristics, and the presence of geometrical imperfections. Suiker et al. [36] also demonstrated the practical applicability of the model for different wall structures fabricated by 3D concrete printing.

This study originally focused on the experimental program and discussion of the test results in terms of buildability. Prediction of the failure mode or numerical resistance for the structures printed in this study could not be performed because the heterogeneous strength and stiffness characteristics of the concrete used in this study, which are necessary to analyze the numerical resistance to failure, could not be estimated from the green strength test. Therefore, numerical predictions of failure modes based on the developed model should be investigated in a future study.

## 4. Mechanical Properties

### 4.1. Specimen Preparation and Testing Methods

In this study, after the printed structures were hardened, the walls of the 3D printed structure in the hardened state from Group 3, as shown in Table 1, were cut into cubic and prismatic specimens for compressive, splitting tensile, and flexural tensile tests. The printed structures were covered by wet burlap until a curing age of 28 days after investigating the buildability of the 3D concrete printed structures, and then they were cut into the cubic and prismatic specimens. Two methods to extract the specimens from the 3D printed structures could be considered based on the time of extraction. In the first method, the specimens are extracted by cutting the 3D printed structures at early ages in the fresh state before the structures reach the final setting period of concrete. The specimens can be easily extracted by cutting or sawing the structures in the early fresh state. However, extracting specimens at early ages can cause hairline cracks in the specimens due to deformation and eventually affect the stability of the specimen shape. In the worst case, the 3D printed structures may collapse when they are cut in the fresh state. In the second method, the specimens are extracted by cutting the 3D printed structures in the hardened state—e.g., by sawing. Sawing specimens in the hardened state can avoid changes in specimen shape and accordingly the deformation of specimens. However, this approach requires extensive skills of the experimental workers to secure the flatness of the cut surface on which loading is applied for the strength test. This method was used in some previous studies [15,18,21,27] and was also adopted in this study. The cut surfaces of some specimens was not sufficiently flat when they were cut from the structures, and such specimens were excluded from the strength test.

**Figure 6:**
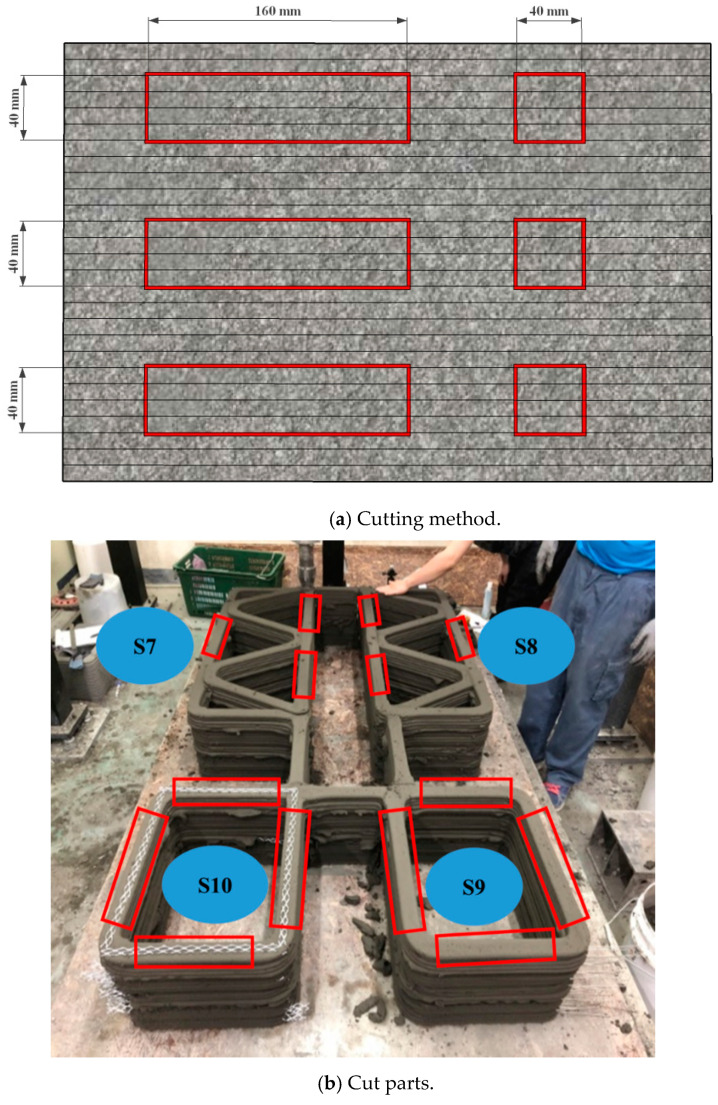
Typical representation of specimen cutting from a 3D printed concrete wall.

A typical representation of specimens cut from a 3D printed concrete wall is shown in Figure 6a. The cubic and prismatic specimens were extracted by saw-cutting three walls of structures S7 and S8 and four walls of structures S9 and S10, as presented in Figure 6b. For specimens cut out of the concrete wall, the dimensions of 40 mm × 40 mm × 40 mm for the cube and 40 mm × 40 mm × 160 mm for the prism were chosen because the bead width of the layers was approximately 40 mm. However, a surface treatment was performed by using an accurate grinder to reduce the influence of the surface conditions because the surface was uneven for the printed specimens. In addition, since the cross-sectional areas of the specimens were variable depending on the bead width and layer height of each specimen, the cross-sectional area was actually calculated by measuring the length of each side of the specimens.

In addition, the monolithic specimens were cast in dimensions of 50 mm × 50 mm × 50 mm for the cubic specimens and 40 mm × 40 mm × 160 mm for the prismatic specimens, as proposed in ASTM C109/C 109M-07 and C348-18 [41,42]. The monolithic specimens were moisture-cured for 24 h after they were cast and then cured in a water tank until a curing age of 28 days was reached.

**Figure 7:**
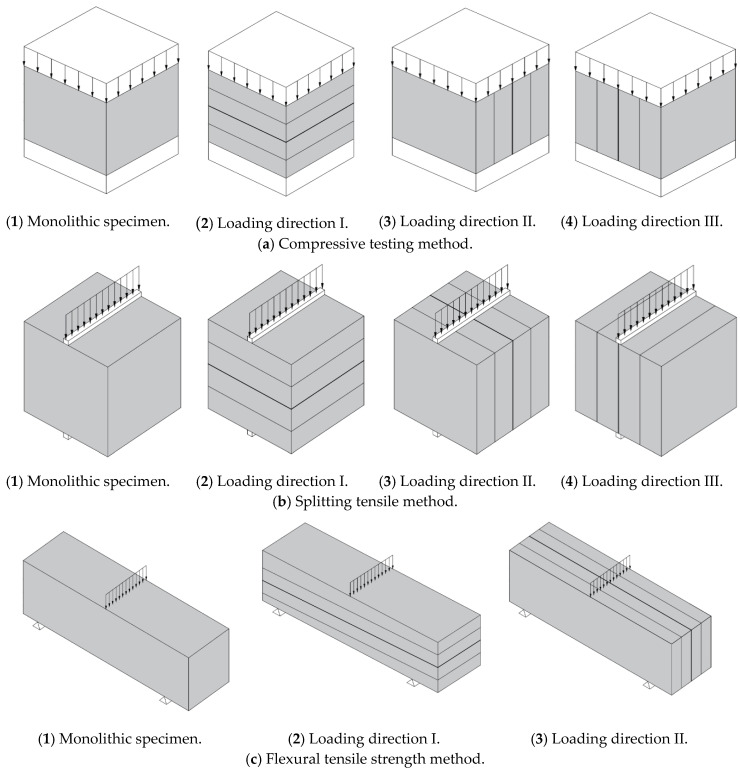
Material testing methods.

Unlike the monolithic specimens, the printed specimens had anisotropic mechanical properties that depended on the different loading directions; this dependency was based on the layer stacking directions of the 3D printed concrete. Accordingly, the printed specimens were tested in three mutually perpendicular loading directions, as shown in Figure 7. The results from the printed specimens tested in the different loading directions were compared to those from the monolithic specimens cast during the printing process of the structures.

The compressive strength test was performed on the cubic specimens. Before the compressive strength testing of the monolithic and printed specimens was performed, the lengths of all sides of the specimens were measured. The compressive strengths of the printed specimens were measured in three loading directions, namely, I, II, and III. However, loading directions II and III were actually the same, and thus, the compressive tests were performed in loading directions I and II. For each loading direction, five monolithic and printed specimens were tested for their compressive strengths in accordance with ASTM C109/C 109M-07 [41]. The compressive strengths of the monolithic and printed specimens were calculated as follows:

(1) $$f_{c}^{\prime }=\frac{P}{l\times b}$$

where *l* is the length (mm), *b* is the width of the specimen (mm), and *P* is the maximum load (N).

The splitting tensile strength test was also determined for the cubic specimens. For the printed specimens, splitting tensile tests were conducted in three loading directions, namely, I, II, and III. For each loading direction, five monolithic and printed cubic specimens were tested for their splitting tensile strengths in accordance with ASTM C 496/C 496M-04 [43]. The splitting tensile strengths were calculated as follows:

(2) $$f_{t}=\frac{2\times P}{\pi \times l\times b}$$

where *l* is the length of line contact of the load (mm), *b* is the nominal cross-sectional dimension (mm), and *P* is the maximum load (N).

For the flexural tensile test, the test in loading direction III could not be performed because of problems encountered while cutting the specimens out of the 3D printed structures. Five prismatic specimens were used for the flexural tensile test in each loading direction (I and II) in accordance with ASTM C348-18 [42]. The flexural tensile strengths of the prismatic specimens were calculated as follows:

(3) $$f_{r}=\frac{3\times P\times l}{2\times b^{3}}$$

where *l* is the distance between the supports (mm), *b* is the length of the squared section of the prism specimen (mm), and *P* is the maximum load (N).

After five specimens were tested for each strength, the means and standard deviations of the test results were calculated to estimate the strength properties of the monolithic and printed specimens.

### 4.2. Compressive Strength

**Figure 8:**
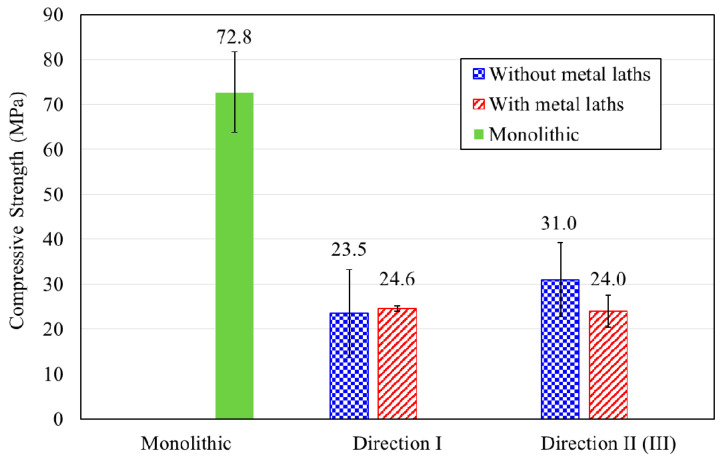
Compressive test results.

Compressive strength tests were performed for both the monolithic and printed cube specimens. The printed specimens were categorized into those without metal lath reinforcement and those reinforced by metal lath reinforcement. The compressive test results are given in Table 3. A comparison between the compressive strengths of the monolithic and printed specimens is also plotted in Figure 8.

The compressive strength of the monolithic specimen was 72.8 MPa, while those of the printed specimens without metal lath reinforcement in loading directions I and II were 23.5 and 31.0 MPa, respectively. For the printed specimens reinforced by the metal lath, the compressive strengths in loading directions I and II were 24.6 and 24.0 MPa, respectively. The compressive strengths of the printed cubic specimens ranged from approximately 32.3 to 42.6% lower than those of the monolithic specimens.

**Figure 9:**
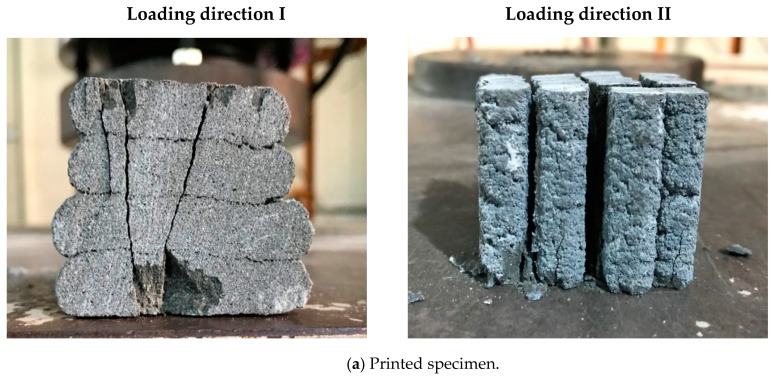

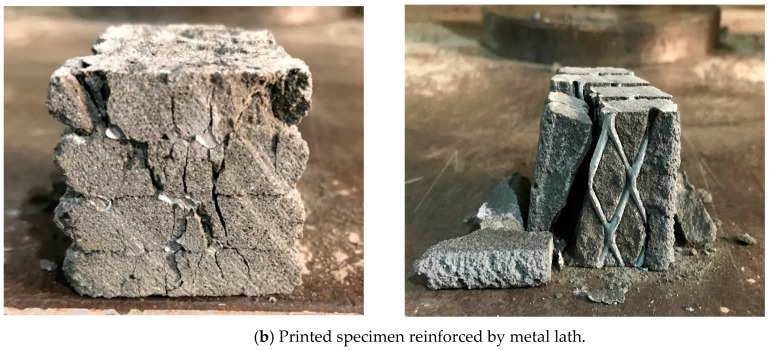
Compressive failure of a printed specimen with and without metal lath in loading directions I and II.

For loading direction I, the compressive strengths of the printed specimens with and without metal lath reinforcement showed similar values. The failure patterns were accompanied by the propagation of vertical cracks over the height of the specimens, as shown in Figure 9. This failure pattern implied that the layer reinforcement in the printed specimens did not affect their compressive strengths in loading direction I.

For loading direction II (or III), the failure of the printed cubic specimens resulted from interface failures between the layers. The failure pattern also indicated that the compressive strengths were highly dependent on the bonding capacity at the interlayers of the printed specimens because the specimens in this loading direction were subjected to lateral deformation under vertical compressive loading.

The compressive strength of the printed specimens without a metal lath was greater than that of the specimens reinforced by a metal lath. This implied that the bonding performance of the printed specimens without a metal lath at the interlayers was higher than that of the printed specimens reinforced by a metal lath at the interlayers. Metal lath reinforcements at the interlayers were expected to improve the bonding and tensile performance of the 3D printed concrete. In contrast, the metal lath reinforcement adversely affected the interlayer adhesion performance due to weak bonding between the reinforcement and concrete filaments. The failure of the printed specimen reinforced by the metal lath shown in Figure 9b actually showed that the printed layer and metal lath reinforcement were separated at the failure interface. This failure pattern shown in the figure supported the compressive strength test results.

### 4.3. Splitting Tensile Strength

**Figure 10:**
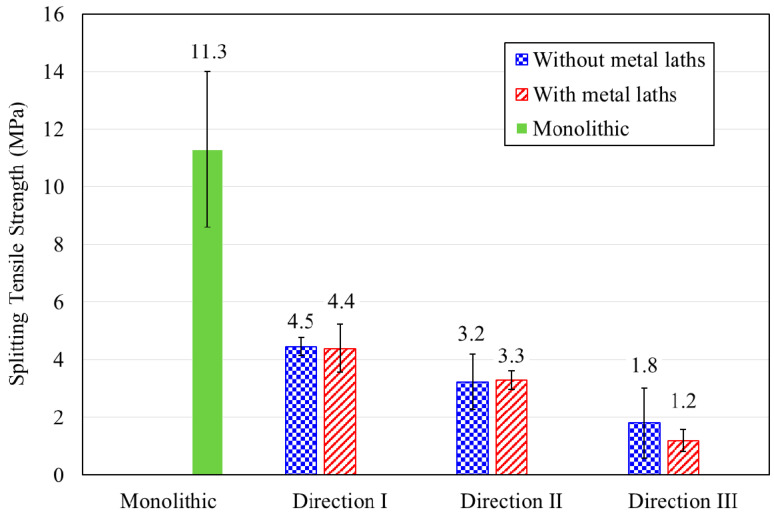
Splitting tensile test results.

Splitting tensile testing was performed on both the monolithic and printed specimens in different loading directions. A comparison of the splitting tensile strengths for the monolithic and printed specimens is shown in Figure 10. The splitting tensile strength of the monolithic specimens was 11.2 MPa, while those of the printed specimens without metal lath reinforcement were 4.5, 3.2, and 1.8 MPa in loading directions I, II, and III, respectively. The splitting tensile strengths of the printed specimens reinforced by metal laths at the interlayers in loading directions I, II, and III were 4.4, 3.3, and 1.2 MPa, respectively. The test results show that the splitting tensile strength of the printed specimens was 11.1~39.7% lower than that of the monolithic specimen. This was because the weak bonding strength at the interlayers led to the low splitting tensile strength of the printed specimens.

**Figure 11:**
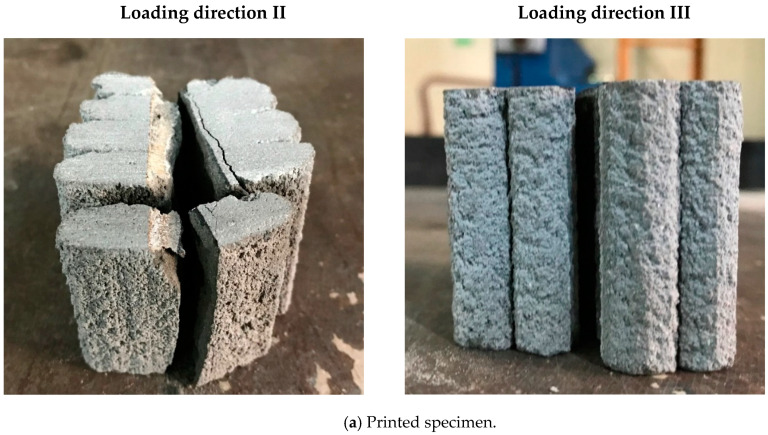

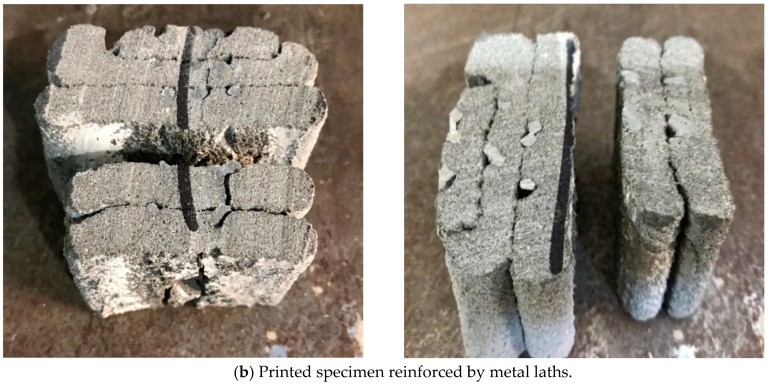
Splitting tensile failure of a printed specimen with and without metal lath in loading directions II and III.

The splitting tensile strengths in loading directions I and II of the printed specimens with and without metal laths were not significantly different. Therefore, the interlayer reinforcement hardly affected the splitting tensile strength, although the metal lath reinforcement at the interlayers was expected to improve the splitting tensile strength of the printed specimens. The failure pattern of the printed specimens in loading direction II is shown in Figure 11. The splitting tensile failure in loading direction II was accompanied by debonding of the interlayers in both the printed specimen without metal lath reinforcement and the printed specimen reinforced by metal laths. This implied that the adhesion between the concrete filaments and reinforcements played an important role and that the imperfect adhesion could not enhance the splitting tensile strength of the printed specimen reinforced by the metal laths compared to that of the printed specimen without the metal laths.

The effect of the imperfect adhesion between the concrete filaments and reinforcements on the tensile strength was also observed in the splitting tensile strength in loading direction III. The splitting tensile strength of the specimens reinforced by the metal laths was lower than that of the specimens without the metal laths. The test results were different from the expectation that the metal lath reinforcement at the interlayer would increase the splitting tensile strength of the printed specimens in this loading direction. The failure pattern of the printed specimens in loading direction III is shown in Figure 11. The splitting tensile failure in loading direction III was also accompanied by debonding of the printed layers in both the printed specimen without metal laths and the printed specimen reinforced by metal laths, such as in loading direction II.

Finally, the test results in this study implied that the bonding conditions between the concrete filaments and reinforcements at the interlayers played a significant role in the splitting tensile behavior. Therefore, the adhesion between the concrete filaments and reinforcements should be confirmed to improve the tensile behavior of 3D printed concrete structures.

### 4.4. Flexural Tensile Strength

**Figure 12:**
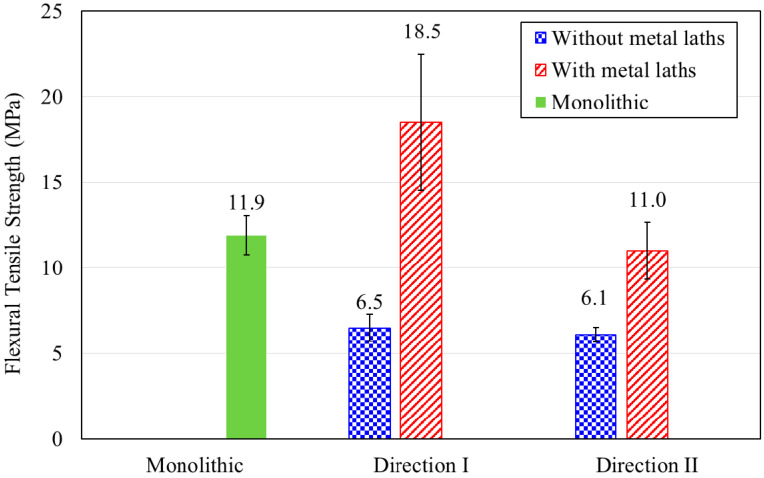
Flexural tensile test results.

The printed prismatic specimens with and without metal laths were tested in two different loading directions, namely, I and II. The flexural strength testing of specimens in loading direction III was not performed because of difficulties in preparing those specimens. A comparison of the flexural tensile strength results for the monolithic and printed specimens is shown in Figure 12.

The flexural tensile strength of the monolithic specimen was 11.9 MPa, while those of the printed specimens without metal lath reinforcement were 6.5 and 6.1 MPa in loading directions I and II, respectively. The test results show that the flexural tensile strengths of the printed specimens without reinforcement were approximately half that of the monolithic cast specimens.

In addition, the flexural tensile strengths of the printed specimens reinforced by metal laths were 18.5 and 11.0 MPa for loading directions I and II, respectively. The flexural tensile strength of the printed specimens without metal lath reinforcement was lower than that of printed specimens reinforced by metal laths. This was because metal lath reinforcement in the tensile zone acted as a tensile reinforcement. Accordingly, the metal lath reinforcement increased the flexural tensile behavior of the 3D printed specimens in both loading directions I and II.

**Figure 13:**
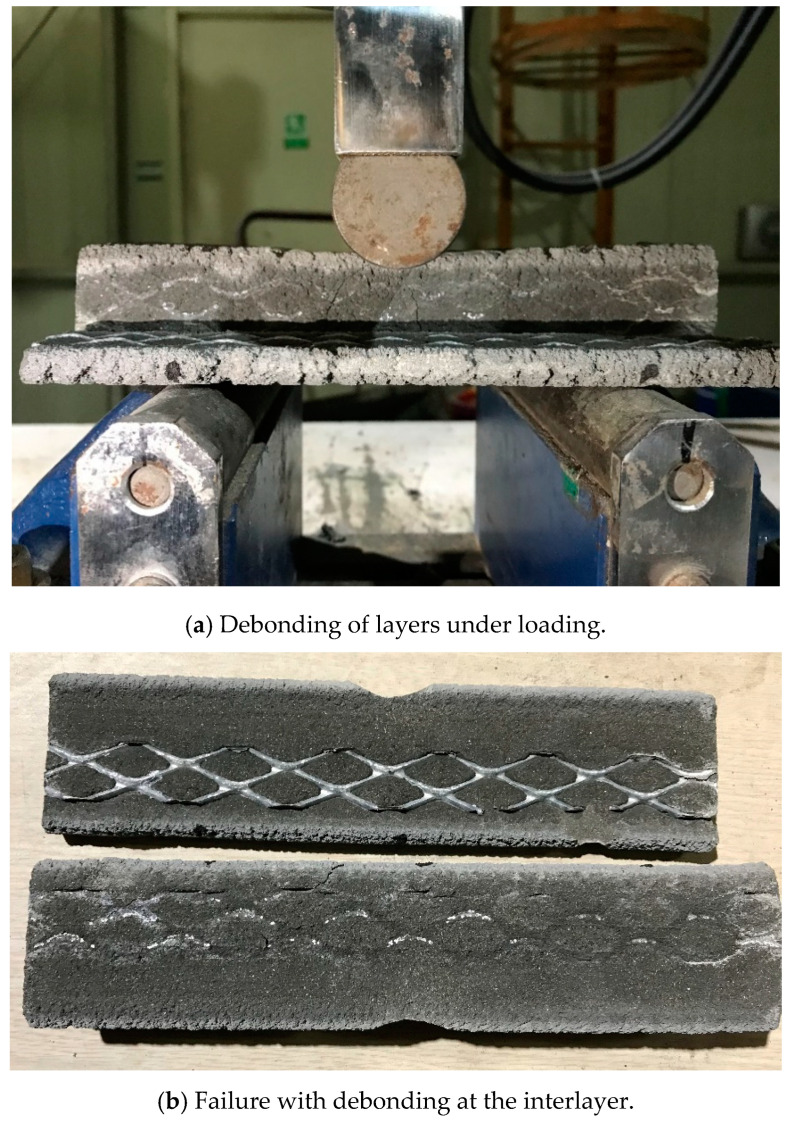
Flexural tensile failure of printed specimens in loading direction II.

However, the flexural tensile behavior in loading direction II, as shown in Figure 13, was accompanied by debonding between the printed interlayers, such as in the case of the splitting tensile behavior described in the previous section. This result indicated that metal lath reinforcement with incomplete interlayer adhesion did not increase the flexural tensile strength as much as expected.

## 5. Conclusions

The buildability and mechanical properties of 3D printed concrete were investigated in this study. Based on the extensive experimental results, the following conclusions can be drawn:

1. The interlayer interval time significantly influenced the buildability of 3D printed concrete. The test results showed that an extended interlayer interval time of up to 300 s contributed to the green strength of the 3D printed concrete and thus increased the buildability of the 3D printed concrete.
2. The 3D printed concrete structures with lateral supports could increase the resistance to collapse due to buckling failure. In particular, a wide connecting width between the lateral support and the structure wall improved the capacity of layer decomposition in the 3D printed concrete.
3. The compressive strengths of the 3D printed cubic specimens were 32.3~42.6% lower than those of the monolithic specimens. In addition, the weak bonding performance between the reinforcement and concrete filaments caused the low compressive strength of the printed specimens in direction II.
4. The splitting tensile strength of the 3D printed concrete specimens was 11.1~39.7% lower than that of the monolithic specimens, depending on the loading directions. The splitting tensile failure was accompanied by debonding between the printed layers, which implied that the bonding performance between the concrete filaments and reinforcements should be confirmed when interlayer reinforcements are included.
5. The flexural tensile strengths of the 3D printed specimens without reinforcements at the interlayers were approximately half that of the monolithic cast specimens. The flexural tensile strength of the printed specimens was improved by the metal lath reinforcements. The failure pattern also revealed that the bonding between the interlayers and reinforcements might influence the flexural strength.

## Figures and Tables

**Table 1:** Details of the printed structures.

Group	Structure Identification	Dimensions	Nozzle Speed (mm/s)	Interlayer Interval Time (s)	RPMs of Mixing Screw (r/m)	Number of Deposition Layers	Ultimate State
Group 1	S1	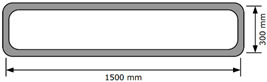	100	36	120	19	Buckling
	S2		80	45	120	49	Buckling
	S3		80	45	140	29 ^(1)^	Tearing of concrete
Group 2	S4	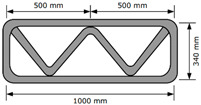	80	55	120	22	Buckling
	S5	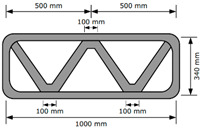	80	57	120	28 ^(1)^	Tearing of concrete
	S6	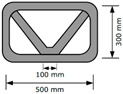	80	30	100	33	Buckling
Group 3	S7	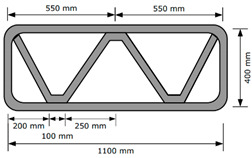	50	300	120	52 ^(1)^	Printing stopped
	S8		50	300	120	52 ^(1)^	Printing stopped
	S9	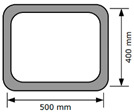	50	300	120	52 ^(1)^	Printing stopped
	S10 ^(2)^	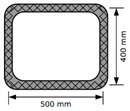	50	300	120	52 ^(1)^	Printing stopped

Note: (1) For the structure, printing stopped at the corresponding layers, and thus, the collapse of the structure was not observed. (2) The structure was reinforced by a metal laths at the interlayers.

**Table 2:** Mixture proportions.

W/B (%)	Unit Weight (kg/m^3^)						
	Water	OPC	SF	FA	Sand	HWRA	Viscosity Agent
0.29	240	576	79	172	1154	8.27	1.65

OPC: Ordinary Portland cement, SF: silica fume, FA: fly ash, and HWRA: high-performance water-reducing agent.

**Table 3:** Test results for the mechanical properties.

Strength	Loading Direction	Monolithic Specimen			Printed Specimen					
					Without Metal Laths			With Metal Laths		
		Number of Specimens	Mean (MPa)	SD (MPa)	Number of Specimens	Mean (MPa)	SD (MPa)	Number of Specimens	Mean (MPa)	SD (MPa)
Compressive strength ($f_{c}^{\prime }$)	Loading direction I	5	72.8	9.0	5	23.5	9.8	5	24.6	0.7
	Loading direction II (III)	5			5	31.0	8.3	5	24.0	3.5
Splitting tensile strength ($f_{t}$)	Loading direction I	5	11.3	2.7	5	4.5	0.3	5	4.4	0.8
	Loading direction II	5			5	3.2	1.0	5	3.3	0.3
	Loading direction III	5			5	1.8	1.2	5	1.2	0.4
Flexural tensile strength ($f_{r}$)	Loading direction I	5	11.9	1.1	5	6.5	0.8	5	18.5	4.0
	Loading direction II	5			5	6.1	0.4	5	11.0	1.7

Note: SD refers to the standard deviation.

## Data Availability

The data used to support the findings of this study are available from the corresponding author upon request.
